# The Forward Effect of Delayed Judgments of Learning Is Influenced by Difficulty in Memory and Category Learning

**DOI:** 10.3390/jintelligence11060101

**Published:** 2023-05-25

**Authors:** Xun Wang, Xinyue Liu, Luyao Chen, Kaiqi Feng, Qun Ye, Haoliang Zhu

**Affiliations:** 1 Intelligent Laboratory of Child and Adolescent Mental Health and Crisis Intervention of Zhejiang Province, School of Psychology, Zhejiang Normal University, Jinhua 321004, China; 2 Key Laboratory of Intelligent Education Technology and Application of Zhejiang Province, Zhejiang Normal University, Jinhua 321004, China; 3 Department of Psychology, Wenzhou University, Wenzhou 325035, China

**Keywords:** delayed JOL, forward effect, memory, category learning, learning strategies

## Abstract

Delayed judgment of learning (JOL) is a widely used metacognitive monitoring strategy that can also enhance learning outcomes. However, the potential benefits of delayed JOL on subsequent learning of new material, known as the forward effect of delayed JOL, and its stability and underlying mechanisms have yet to be fully explored. In this study, we investigated the forward effect of delayed JOL using previously unexamined word pair materials and explored the boundary conditions of this effect by manipulating the difficulty of the materials. We also examined this effect within the context of category learning. Our findings demonstrate that delayed JOL significantly enhanced the retention of new information (Experiment 1A), while the forward effect of the delayed JOL occurred only for material with a certain degree of difficulty rather than for easy material (Experiment 1B). These findings were extended and replicated using category learning (Experiment 2). These results suggest that delayed JOL can be used as a preparation strategy for subsequent learning, particularly when faced with challenging materials. Our study provides novel insights into the potential benefits and limitations of delayed JOL and contributes to our understanding of the underlying mechanisms that govern metacognitive monitoring and learning strategies.

## 1. Introduction

Judgment of learning (JOL) is a predictive judgment about the performance of a learned item in subsequent tests (Nelson and Dunlosky 1991). As an important form of metacognitive monitoring, the accuracy of JOL plays an essential role in determining whether individuals can learn efficiently (Koriat et al. 2006; Metcalfe and Finn 2008; Metcalfe 2009). Furthermore, researchers have found that metacognitive monitoring performance is more accurate when JOLs are made at an interval after learning (e.g., 30 s of studying other items), compared with immediate JOL, known as the delayed JOL effect (Dunlosky and Nelson 1997; Koriat and Ma’ayan 2005; Son and Metcalfe 2005; Rhodes and Tauber 2011; Kelley et al. 2021). Additionally, studies also have found that delayed JOL not only improves the accuracy of metamemory monitoring, but also improves the recall performance of the judged items (Sikström and Jönsson 2005; Serra and Dunlosky 2006; Jönsson et al. 2012; Sundqvist et al. 2012; Myers et al. 2020; Li et al. 2022). Jönsson et al. (2012) suggested that this facilitation is related to the covert retrieval attempts of the target information resulting from the delayed JOL, which itself facilitates learning and is often referred to as the retrieval practice effect (McDaniel et al. 2007; Karpicke and Roediger 2008; Carpenter et al. 2008; Hausman and Rhodes 2018). In other words, when individuals make metacognitive judgments, they engage in covert retrieval attempts to predict their own test performance (Kubik et al. 2022). Thus, compared to restudy, one of the most common learning strategies without any item retrieval, delayed JOL can more effectively promote learners’ memory for learned items.

The current research focuses on the forward effects of delayed JOL, which are receiving increasing attention as an indirect but future-oriented benefit of delayed JOL. Recent studies have found that delayed JOL not only promotes the retention of previously learned information, but also has a positive effect on the subsequent learning of new material (Lee and Ha 2019; Kubik et al. 2022). To examine the forward effect of JOL, methods were developed based on the paradigm of the forward test effect (FTE). In this approach, the material to be memorized is divided into several separate learning blocks (e.g., Szpunar et al. 2008; Sohlberg et al. 2021; Kubik et al. 2022), and participants are divided into at least two groups: a delayed JOL group and a control group. The JOL group undergoes the metacognitive assessment after each individual study block, while the control group often restudies the content a second time. Both groups then take a test on the final study block. The results of the interim test in the final learning block for both groups demonstrates the effect of forward effect of JOL. The difference between the two groups in terms of the results achieved in the final block is likely due to the JOL group making learning judgements in the previous block, resulting in the forward effect.

There are several possible explanations for the forward effect of delayed JOL. On the one hand, the reasons may be similar to the forward test effect (for an overview, see Yang et al. 2018). Context change theory emphasizes the role of contextual change induced by an interim task. This theory postulates that contextual changes between learning items enhance individual learning by reducing proactive interference and increasing learning engagement (Szpunar et al. 2008; Bäuml and Kliegl 2013; Sohlberg et al. 2021). A recent study by Kubik et al. (2022) demonstrated that delaying JOL using an interim task inserted during learning multiple lists attenuated the number of intrusions from prior lists. On the other hand, various forms of metacognitive judgments have been shown to impact learners’ future learning behaviors. For example, the JOL group requires learners to consider their own learning status after a learning process. This monitoring and evaluation of their learning behavior encourages learners to adjust their learning efforts by changing their study behavior to optimize subsequent learning, such as allocating study time, selecting restudy materials, and adjusting learning strategies (Sahakyan et al. 2004; Kornell and Metcalfe 2006; Metcalfe and Finn 2008; Mitchum et al. 2016; Wang et al. 2020). In studies on the forward effect, interim tasks inserted during the learning of multiple lists are thought to have metacognitive benefits that can prompt learners to adopt more effective coding strategies during subsequent learning (Cho et al. 2017; Lee and Ahn 2018). Lee and Ha (2019) found that even when learners were not given the freedom to choose what they study and for how long, the mere act of making metacognitive judgments directly facilitated the learners’ subsequent learning of new material.

However, not all acts of making JOLs will produce a facilitative forward effect. Kubik et al. (2022) used single German words as experimental material and examined the forward effects of delayed JOLs with word stems (e.g., ele_____?) versus JOLs with complete words (e.g., elephant), in comparison to restudy and retrieval practice. Participants consecutively studied five lists of 20 words with the goal of recalling as many of them as possible on a final cumulative recall test. After the presentation of each of the first four lists, the participants either restudied the list, made JOLs with complete words, made JOLs with word stems, or were tested on word stems. The results showed that practicing retrieval and making JOLs with word stems, but not making JOLs with complete words, facilitated interim recall performance of the last list compared to restudy. This suggests that creating JOLs with incomplete information can promote new learning, perhaps because it elicits covert retrieval attempts, but making JOLs with complete information cannot. Similarly, in another related study, Lee and Ha (2019) examined whether the act of making judgments of learning (JOLs) on prior material contributed to the learning of new material in the category learning of painters’ styles in three experiments. The results showed that the act of making category-level JOLs and global metacognitive judgments facilitated the learning of new material. However, it is worth noting that simply asking learners to make item-level JOLs did not facilitate learning compared to restudy. One explanation for the lack of forward effects in these item-level JOLs is that they required learners to evaluate their learning about specific painting–artist pairs and thus did not meet the learning goal of abstracting the artist’s general painting style from the studied examples. However, as Lee and Ha pointed out, another explanation is that the participants were unable to adequately evaluate their learning due to the simultaneous presentation of cues (paintings) and target items (artist names). Simultaneous presentation may place learners in a restudy-like state, thereby reducing the probability that learners will engage in covert retrieval practice.

Given that only a few studies have explored the future-oriented benefit of delayed JOL, the purpose of the present study was to replicate and extend this work on the forward effect of item-based JOL to word pair memory and category learning tasks, and to further explore the boundary conditions of this effect. We pursued three main research questions. First, we examined whether the same forward effect of delayed JOL could be observed when we used typically associated word pairs rather than single words as experimental material. To our knowledge, no previous study has used associated word pairs (e.g., dog–cat) to explore the forward effect of delayed JOL. If this effect is robust, we hypothesize that cue-only delayed JOL (the first word in a word pair, e.g., dog) might similarly facilitate learners’ performance on subsequent material, since this process also involves covert retrieval attempts for the missing target word. In the present experiments, we examined whether delayed JOLs affect subsequent learning by comparing them to restudy, which serves as a common and neutral control condition that does not involve any overt or covert retrieval practice, ensuring that the two conditions differed only in the behavior of performing delayed JOLs.

As a second goal, we explored whether the item-level delayed JOL could similarly improve the learning efficiency of new category material. In a real-life educational context, what is important to learn often goes beyond memorizing specific items. Students also need to be able to generalize what they have learned from specific examples to other examples in a particular category, that is, category learning (Smith et al. 2010; Lee and Ahn 2018). Different from item learning, inductive learning is a process where learners are required to abstract rules from a set of exemplars (Yang et al. 2019). For example, if students have mastered Van Gogh’s painting style in art class, they will later classify a painting that is unfamiliar but fits that style as Van Gogh’s work. Likewise, if a teacher has shown some examples of swallows in a biology class, then when students see a small blue–black bird with pointed wings and a forked tail in real life, they will be able to classify it as a swallow. Although Lee and Ha (2019) were the first to explore the role that delayed JOL plays in category learning, it remains unclear whether item-based delayed JOL can facilitate subsequent learning of new categories, and we do not know whether the ineffectiveness of the forward effect is due to a mismatch between item-based JOLs and category learning goals, or if it is due to the lack of a retrieval practice process when making JOLs. Therefore, we made some adjustments based on Lee and Ha (2019) to achieve this goal. First, we used cue-only JOLs rather than cue-target JOL, thus enabling learners to make retrieval attempts. Second, we used pictures of artificial conceptual animals as learning materials, which effectively excluded the influence of learners’ prior experience compared to materials for learning drawing styles. Third, we further manipulated the difficulty level of the category learning material to determine whether there were boundary conditions for the forward effects of delayed JOL.

Material difficulty, as an important factor influencing learner performance after retrieval, is the third focus of the current study. Previous studies have found that retrieving materials of different difficulty levels leads to different memory performances. For example, Pyc and Rawson (2009) suggested that memory performance after successfully retrieving items with a higher difficulty is better than for items with lower difficulties. According to the strength and retrieval effort theory, the enhancement of knowledge is proportional to the effort of retrieval, and the key to the retrieval practice effect lies in the retrieval effort. Compared with simple retrieval activities, learners will invest more effort in retrieving difficult materials, which will help to produce better learning results (Bjork 1975; Jacoby et al. 1978; Pyc and Rawson 2009; Kliegl et al. 2019). Then, in the delayed JOL condition involving covert retrieval, does the difficulty of the material also have a different impact on the learning effect? More importantly, how does material difficulty affect the forward effect? Compared with the direct influence of retrieval practice on “learned” materials, the forward effect focuses on its indirect promotion and the transfer of subsequent “unlearned” materials. In this process, the learner’s effort and other motivational factors may play a more significant role (Yang et al. 2018). Difficult items may motivate learners to make a greater effort to retrieve and code new content due to the difficulty or failure of retrieval (Cho et al. 2017). To explore this, we introduced an independent variable of learning material difficulty by manipulating the correlation of word pairs and feature dimensions during category learning, expecting that the forward effect of delayed JOL might be more advantageous in more difficult materials.

In summary, three experiments were designed to explore the questions posed above. Experiment 1A focused on determining whether the forward effect of delayed JOL occurs during encoding of memories of associated word pairs by comparing the delayed JOL and restudy group, while Experiment 1B further refined the level of difficulty of the memorized material based on Experiment 1A to explore the role that material difficulty played in this process. Experiment 2 then examined whether it was possible to replicate Experiment 1 when the task was extended from the memory domain to the category learning task.

## 2. Experiment 1A

### 2.1. Materials and Methods

#### 2.1.1. Participants

We used G*Power3.1.9 to calculate the required sample sizes based on previous studies that reported effect sizes ranging from 0.93 to 1.33 (Lee and Ahn 2018; Yang and Shanks 2018). Our initial calculations indicated that to observe a significant (two-tailed, α = 0.05) forward effect at 0.80 power, we would need 10–20 participants per condition. However, given the complex nature of factor analysis and the potential for unforeseen issues (MacCallum et al. 1999), we adopted a more conservative approach and increased the sample size to approximately 30 participants for each condition. A total of 64 undergraduates were recruited from the Zhejiang Normal University (ZJNU) participant pool, then randomly assigned to the delayed JOL and restudy conditions (n = 32 in each group). Three participants were excluded from the final statistical analysis due to their low recall performance and failure to adhere to the experimental instructions. Specifically, two of them had a recall rate below 15%, which was lower than the 2.58 standard deviation of the group, while the third participant did not provide the necessary responses as instructed. The valid sample consisted of 61 participants with a mean age of 19.79 years (SD = 1.42) and 41 (67%) females. The participants gave informed consent and obtained a corresponding reward after the experiment.

#### 2.1.2. Materials

Word pairs, each consisting of a cue word and a target word, were used in this experiment. In order to avoid the ceiling effect and floor effect caused by materials that are too simple or too difficult (Akdoğan et al. 2016). Chinese two-character word pairs of medium difficulty were selected as experimental materials in this experiment (for details, see Supplementary Materials, Table S1).

Firstly, 200 two-character words were selected from the Modern Chinese Frequency Dictionary and randomly combined into 100-word pairs in the form of “Yu Mi (cue word)—Gao Shan (target word)”. All of the words are emotionally neutral, the word frequency interval was controlled between 0.00076 and 0.00997, the number of syllables was kept between 4 and 6, the total number of strokes was set between 7~30, and word pair was formed randomly (Yu et al. 2020). The difficulty of word pairs was determined by the degree of semantic association between the cue word and the target word. Furthermore, we recruited 30 college students to rate the semantic relatedness of word pairs on a 7-point scale, with 1 representing no relationship and 7 representing a very close relationship. Then, we calculated the average correlation degrees of each word pair and ranked them. Finally, 42 word pairs of medium difficulty (3.34 ± 1.1) were selected. Among them, 36 pairs were randomly divided into three lists, with 12 pairs for each formal experiments. The remaining 6 pairs were used in the practice phase, and the results were not included in the final analysis.

#### 2.1.3. Design and Procedure

The learning strategy (Delayed JOL vs. Restudy) was manipulated between participants as the only independent variable. The entire study was administered on a computer using E-Prime 2.0 in an individual, sound-proof testing room.

A simplified version of the multiple-list learning paradigm was used in this experiment to examine the forward effect of JOL. Before starting the experiment, participants in both groups were told that they would study three lists of double-word pairs. After studying each list, the computer randomly decided whether their interim task would be to make a JOL or to study the list again. However, it should be noted that the task decisions were predetermined and not randomized. The interim task for the delayed JOL group was to make a JOL, while the restudy group’s task was to restudy after the first two lists. The task for both groups after the third list was to complete the test. The participants were also told that there would be a cumulative test at the end of the study.

During the experiment, the participants first learned 12 cue–target word pairs in List 1, and each word pair was presented for 5 s. Second, the participants were asked to perform simple math calculations (799 minus 3, consecutively) for one minute as a distraction task. Then, participants in the two groups completed different interim tasks. For the delayed JOL group, the participants made cue-only JOLs on the word pairs they had just learned one by one, and the judgment time for each word pair was 5 s. The participants were required to predict the probability that they could recall the right word when the left word appeared in the subsequent test, and the probability gradually increased from 1 to 6. For the control group, the participants were asked to restudy the word pairs they had just studied in a random order, again for 5 s each. Then, the two groups followed the same procedure for the study of List 2.

**Figure 1:**
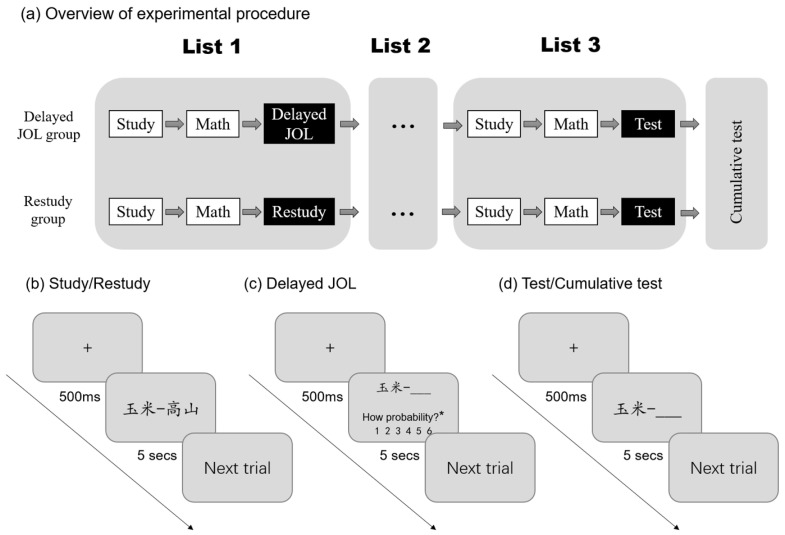
Experimental procedure for exploring the forward effect of delayed JOL. (**a**) Overview of the experimental procedure. The delayed JOL group made JOLs after studying List 1 and List 2 and completed an interim test in the final block. The restudy group restudied each previously studied block except the final one, then took an interim test in the final block. All groups completed a final, cumulative test following the interim test. (**b**) Example of the process for study or restudy; (**c**) example of the process for delayed JOL; (**d**) example of the process for a test or cumulative test. The asterisk indicates that the sentence has been abbreviated for better visualization. In the actual experiment, the full sentence of “How probability?” was “How probability do you think you will be able to correctly recall the word on the right in the following test?”

List 3 is the target list or key list. The participants learned the word pairs and completed a one-minute calculation task. Then, both groups completed an interim test in which only the cue word was presented, and the participants were asked to recall the target word within 5 s. If the delayed JOL group’s test performance in the target list is better than that of the restudy group, it is believed that making a JOL on previously learned materials can promote the learning of subsequent new materials; that is, there is a forward effect of delayed JOL. Finally, all participants completed a cumulative test of the three lists, with all word pairs presented in random order. There was no feedback in the interim and cumulative tests, and the participants were allowed not to respond if they forgot the target word (see Figure 1).

### 2.2. Results

**Figure 2:**
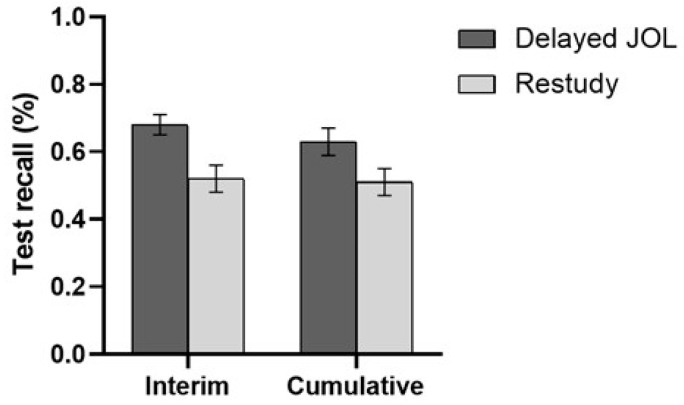
Percentage of correct responses on the interim test and cumulative test in Experiment 1A. Error bars represent 1 standard error of the mean.

Figure 2 shows the average proportion of correct answers under each experimental condition. An independent sample *t*-test was used to analyze the target list recall scores of the two groups. Not surprisingly, there was a significant main effect of the learning strategy, and participants in the delayed JOL group (*M* = 0.68, *SD* = 0.18) recalled a significantly higher proportion of target words in the target list than those in the restudy group (*M* = 0.52, *SD* = 0.21), *t*(59) = 3.32, *p* = .002, *d* = 0.85. In addition, although the results of cumulative testing were not our primary concern, we calculated the average cumulative test recall for both groups. We found that the main effect of the learning strategy was also significant, and the cumulative recall performance of the delayed JOL group (*M* = 0.63, *SD* = 0.21) was significantly higher than the restudy group (*M* = 0.51, *SD* = 0.20), *t*(59) = 2.28, *p* = .027, *d* = 0.58.

### 2.3. Discussion

Previous studies have shown a forward effect of delayed JOL in single-word learning. The present study replicated this concept and demonstrated that this forward effect was also present during the learning of associative word pairs. The results of Experiment 1A showed that participants who made JOLs while learning previous study lists (i.e., Lists 1–2) were better able to learn and retain a new study list (i.e., List 3) relative to participants who had not previously made JOLs; that is, the cue-only delayed JOL dose had a forward effect, which was consistent with the findings of Kubik et al. (2022). In addition, Experiment 1A also compared the cumulative test performance and found that the memory of the delayed JOL group was significantly better than that of the restudy group. This suggests that delaying JOL enhances memory retention for both “old” and “new” items, which is consistent with previous findings (Jönsson et al. 2012; Putnam and Roediger 2013; Akdoğan et al. 2016; Li et al. 2022).

## 3. Experiment 1B

In Experiment 1A, the forward effect of delayed JOL was confirmed using word pairs of medium difficulty, which had certain correlations but were still difficult to remember. However, we wanted to further explore whether the failure to retrieve the previous list caused the learner to put more effort into learning the subsequent difficult material. Thus, based on Experiment 1A, the purpose of Experiment 1B was to investigate the effect of material difficulty on the forward effect of delayed JOL. We hypothesized that the target list performance of the delayed JOL group would be better than that of the control group for the low correlation word pairs, while there would be no difference for the high correlation word pairs.

### 3.1. Materials and Methods

#### 3.1.1. Participants

We conducted a priori sample size calculations using G*Power3.1.9 to determine a reliable forward effect with an α = 0.05, a power of 1−β = 0.80, and an effect size of *d* = 0.74. These calculations indicated that a sample size of n = 30 per group (total N = 60) would be sufficient. Sixty participants (44 females; *M*_age_ = 20.44, *SD*_age_ = 1.32) were recruited from the ZJNU participant pool and were randomly divided into the delayed JOL and restudy conditions (n = 30 in each group). The participants gave informed consent and received a corresponding reward after the experiment.

#### 3.1.2. Materials

A total of 42 cue–target word pairs were selected (for details, see Supplementary Materials, Table S2). Among them, 36 pairs were used for formal experiments and were randomly divided into 3 lists, each consisting of 6 high-related word pairs and 6 low-related word pairs. The remaining 6 pairs were used in the practice phase, and the results were not included in the final analysis. All the materials were assessed in advance, and there was a significant difference in difficulty between high-related word pairs (4.36 ≤ M ≤ 5.80) and low-related word pairs (1.14 ≤ M ≤ 1.78).

#### 3.1.3. Design and Procedure

This experiment employed a 2 (Learning strategy: delayed JOL vs. restudy) × 2 (Material difficulty: high-related vs. low-related) mixed design, with learning strategy used as a between-subjects variable and material difficulty as a within-subjects variable. Experiment 1B also adopted the multi-list learning paradigm. Except for the difficulty of word pairs (6 high-related word pairs and 6 low-related word pairs were randomly presented), the process was the same as 1A.

### 3.2. Results

**Figure 3:**
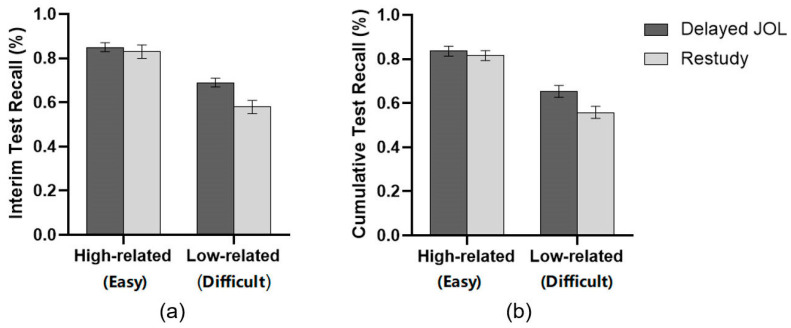
(**a**) List 3 interim test recall; (**b**) cumulative test recall. Error bars represent 1 standard error of the mean.

For the recall of the target list, a 2 (Learning strategy: Delayed JOL vs. Restudy) × 2 (Material difficulty: high-related vs. low-related) two-factor ANOVA revealed no significant difference between the delayed JOL group (*M* = 0.77, *SD* = 0.25) and restudy group (*M* = 0.71, *SD* = 0.25), *F*(1, 58) = 3.564, *p* = .064, $\eta_{p}^{2}$ = 0.058. However, there was a significant main effect of material difficulty, indicating that the target list recall of high-related word pairs (*M* = 0.84, *SD* = 0.13) was significantly higher than that of low-related word pairs (*M* = 0.63, *SD* = 0.17), *F*(1, 58) = 185.245, *p* < .001, $\eta_{p}^{2}$ = 0.762. Moreover, the analysis revealed a significant interaction between learning strategy and material difficulty, *F*(1, 58) = 8.479, *p* = .005, $\eta_{p}^{2}$ = 0.128. Further simple effect analysis showed that there was no significant difference between the delayed JOL group (*M* = 0.85, *SD* = 0.13) and the restudy group (*M* = 0.83, *SD* = 0.14) for high-related word pairs, *F*(1, 58) = 0.396, *p* = .531, $\eta_{p}^{2}$ = 0.007. However, for low-related word pairs, the delayed JOL group (*M* = 0.69, *SD* = 0.16) performed significantly better than the restudy group (*M* = 0.58, *SD* = 0.17), *F*(1, 58) = 6.978, *p* = .011, $\eta_{p}^{2}$ = 0.107 (see Figure 3).

A similar ANOVA on cumulative test recall revealed a significant main effect on material difficulty, *F* (1, 58) = 202.353, *p* < .001, $\eta_{p}^{2}$ = 0.777, indicating better memory for high-related word pairs (*M* = 0.83, *SD* = 0.13) and worse memory for low-related word pairs (*M* = 0.61, *SD* = 0.15). Moreover, significant interactions were found between learning strategy and material difficulty, *F* (1, 58) = 5.835, *p* = .019, $\eta_{p}^{2}$ = 0.091. Further simple effect analysis showed that there was no significant difference between the delayed JOL group (*M* = 0.84, *SD* = 0.11) and the restudy group (*M* = 0.82, *SD* = 0.14) for high-related word pairs, *F* (1, 58) = 0.355, *p* = .554, $\eta_{p}^{2}$ = 0.006. However, for low-related word pairs, the delayed JOL group (*M* = 0.65, *SD* = 0.15) performed significantly better than the restudy group (*M* = 0.56, *SD* = 0.15), *F* (1, 58) = 6.153, *p* = .016, $\eta_{p}^{2}$ = 0.096. No main effect was observed on learning strategy, *F* (1, 58) = 3.166, *p* = .08, $\eta_{p}^{2}$ = 0.052.

### 3.3. Discussion

Experiment 1B explored the effect of material difficulty on the forward effect of delayed JOL by manipulating the correlation of word pairs at the memory task level, which had not been previously explored. Firstly, for the target list recall performance, whether under the condition of delayed JOL or restudy, the recall performance of high-related word pairs was significantly better than that of low-related word pairs, indicating that the experimental materials we selected were reliable. Secondly, the interaction between the learning strategy and material difficulty was significant, with the delayed JOL group performing significantly better than the restudy group for low-related word pairs, but no significant difference for high-related word pairs. This suggests that the forward effect of delayed JOL was only present in materials with some difficulty compared to materials that were very easy to learn. This result is in line with our hypothesis and consistent with previous related studies (Pastötter et al. 2013; Yang et al. 2022). However, in Experiment 1B, although the delayed JOL group showed better memory performance than the restudy group, the difference in retention between the two groups did not reach statistical significance. This may have been due to the null effect of the study strategy on easy items, which attenuates the overall main effect. In other words, the lack of a forward effect for easy materials resulted in a lower overall forward effect, which masked the main effect of the learning strategy.

For cumulative test performance, there was also a significant interaction between the learning strategy and material difficulty, which demonstrated that there was no significant difference between the delayed JOL group and the restudy group on the high-related word pairs. In contrast, the delayed JOL group performed significantly better than the restudy group on the low-related word pairs. This demonstrates that, under the multiple-list learning paradigm, the facilitation effect of delayed JOL were more evident in difficult material, whether for new items or learned items.

## 4. Experiment 2

Experiments 1A and 1B demonstrated that delayed JOL promoted the learning of subsequent new materials at the level of a memory task, and that this promoting effect was more advantageous in medium and difficult materials. In Experiment 2, we extended the forward effect of delayed JOL from the memory domain to category learning. Since previous studies have not found the forward effect of item-based JOLs on category learning, we expected different experimental results in the present study by making a small change, e.g., replacing the JOL that provided complete information with the JOL that provided only the cue item. We hypothesized that (1) the forward effect of delayed JOL also existed in the categorical learning task, and (2) the material difficulty affects the forward effect; that is, the target list score of the delayed JOL group would be better than that of the restudy group in the more difficult categorical material.

### 4.1. Materials and Methods

#### 4.1.1. Participants

We conducted a priori sample size calculations using G*Power3.1.9 to determine a reliable forward effect with an α = 0.05, a power of 1−β = 0.80, and an effect size of *d* = 0.74. These calculations indicated that a sample size of n = 30 per group (total N = 60) would be sufficient. Sixty participants (46 females; *M*_age_ = 19.84, *SD*_age_ = 2.02) were recruited from the ZJNU participant pool and were randomly divided into the delayed JOL and restudy conditions. The participants gave informed consent and received a monetary reward as compensation for participating.

#### 4.1.2. Materials

The materials used in this experiment were artificial animals designed according to artificial concepts, and the material design refers to the experimental materials of (Minda and Ross 2004). The difficulty of the classification task is mainly reflected in the number of feature dimensions, and the variation in the number of feature dimensions affects the learning outcomes (Maddox et al. 2004). Thus, our difficult category materials for this study were animals consisting of 6 dimensions, each with 3 levels: head shape (round, square, triangle), antennae (upward, backward, forward), body (vertical stripes, horizontal stripes, grid stripes), tail (horizontal, zigzag, wavy), eyes (open, closed, half-closed), and mouth (upward, horizontal, downward). The simple category materials included animals composed of 4 dimensions, and each dimension includes 3 levels: head shape (round, square, triangle), antennae (upward, backward, forward), body (vertical stripes, horizontal stripes, grid stripes), and tail (horizontal, zigzag, wavy).

**Figure 4:**
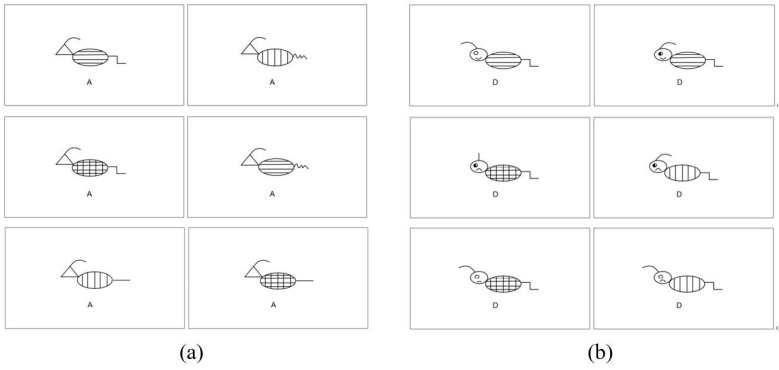
(**a**) An example of a simple category in List 1. Their common features include triangular heads and backward antennae. (**b**) An example of a difficult category in List 1. Their common features include rounded heads and zigzag tails.

A total of 12 categories were created and randomly assigned to three lists. Each list consisted of four categories (A/B/C/D), of which two were categorized as difficult and the other two as easy. Each category was comprised of 6 examples, resulting in 24 examples per list and a total of 72 examples. In addition, two categories were made as materials for practice experiments. Artificial animals were classified according to their common features. An easy category indicated that two of the four characteristics were common, while a difficult category required learners to generalize two common characteristics from the six dimensions. The specific common characteristics of each category were as follows: (1) A1: antennae backward, triangular head; B1: horizontal striped body, horizontal tail; C1: antennae forward, eyes closed; D1: round head, zigzag tail; (2) A2: round head, wavy tail; B2: antennae up, grid striped body; C2: horizontal striped body, eyes open; D2: antennae backward, horizontal tail; (3) A3: antennae backward, horizontal tail; B3: vertical striped body, wavy tail; C3: mouth up, grid striped body; D3: square head, mouth down. Figure 4 shows examples of two of these categories (for details, see Supplementary Materials, Figure S1).

#### 4.1.3. Design and Procedure

This experiment employed a 2 (Learning strategy: delayed JOL vs. restudy) × 2 (Material difficulty: difficult category vs. easy category) mixed design, with learning strategy as a between-subjects variable and material difficulty as a within-subjects variable. The entire study was administered on a computer using E-Prime 2.0.

Prior to the formal experiment, participants completed a practice phase in which they learned two categories of artificial animal pictures (one easy and one difficult) and then completed a one-minute distraction task. The participants were informed that the formal experiment would include three interim tasks (restudy, delayed JOL, and testing), and were given the opportunity to experience each task. During the practice phase, the participants were able to ask the experimenter for clarification if they were confused about any aspect of the experimental procedure. Once they completed the practice phase and fully understood the experimental procedure, they proceeded to the formal experiment. During the formal experiment, the experimenters did not provide any assistance to the participants.

In the formal experiment, the participants were required to learn a total of 12 categories from three lists. They were informed that for each list, they would have to perform an interim task (delayed JOL or restudy), which was randomly determined by the computer. However, in fact, the interim task for each list was predetermined. The participants in the delayed JOL group were given the interim task of JOL after studying the first two lists, while the participants in the control group were asked to restudy. However, the task for both groups was a test after studying the last list.

**Figure 5:**
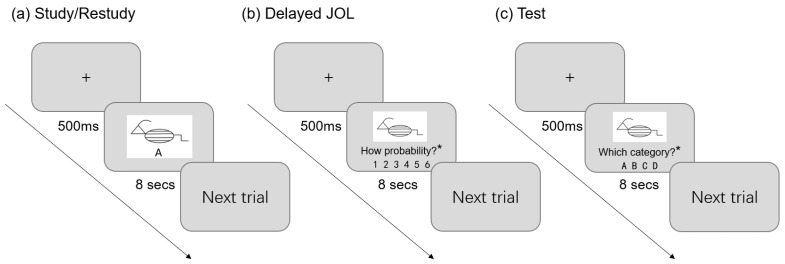
(**a**) Example of the process for study or restudy; (**b**) example of the process for delayed JOL; (**c**) example of the process for the test. The asterisk indicates that the sentence has been abbreviated for better visualization. In the actual experiment, the full sentence of “How probability?” was “How probability do you think you will be able to correctly identify the species of the animal in the following test?” Similarly, “Which category?” means “Which category do you think the animal belongs to?”

Specifically, during the learning process of each list, the participants needed to learn a total of 4 categories, including 2 easy categories and 2 difficult categories, and each category had 6 sample pictures. The sample pictures and their names were presented simultaneously for 8 s, and the samples for each category were presented in a concentrated manner. Then, after being asked to complete a one-minute distraction task (799 minus 3, consecutively), the participants were prompted by the computer to complete an interim task. In the JOL task, the participants were randomly presented with 24 sample images without names. They were asked to predict the probability that they would be able to classify the images correctly within 8 s, and the probability gradually increased from 1 to 6. The restudy task involved studying all the samples of the four categories again. For the test task of the final list, 24 sample pictures were randomly presented with four category names, ABCD, and the participants were required to select the correct category name for each sample within 8 s. For example, if the participants successfully generalized the common features of category A animals during learning, they would be able to classify all animals with triangular heads and backward antennas as A during testing (see Figure 5).

### 4.2. Results

**Figure 6:**
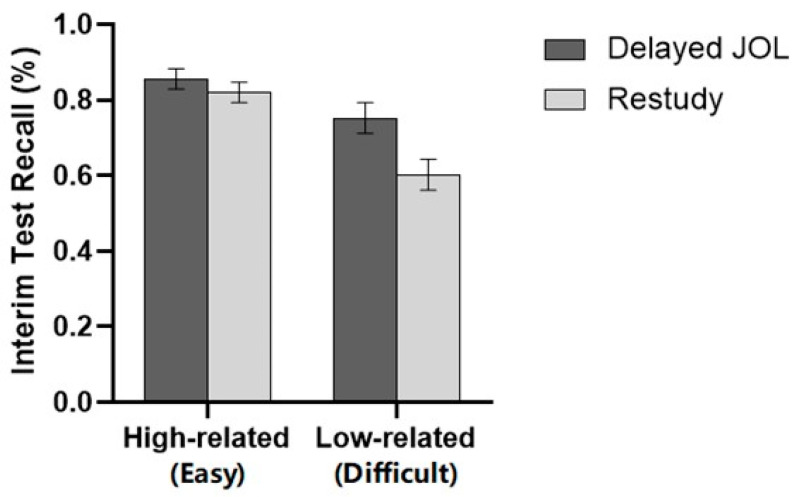
Correct proportion on the interim test for each group in Experiment 2. Error bars represent 1 standard error of the mean.

For the performance of List 3, a 2 (Learning strategy: delayed JOL vs. restudy) × 2 (Material difficulty: difficult category vs. easy category) two-factor ANOVA revealed a significant main effect on learning strategy, *F* (1, 58) = 4.683, *p* = .035, $\eta_{p}^{2}$ = 0.075. Participants who made delayed JOL (*M* = 0.80, *SD* = 0.16) as the interim task performed significantly better than those who simply repeated the study (*M* = 0.71, *SD* = 0.18). The ANOVA also showed a significant main effect of material difficulty, *F* (1, 58) = 51.145, *p* < .001, $\eta_{p}^{2}$ = 0.469. On average, the target list score of simple category materials (*M* = 0.84, *SD* = 0.15) was significantly higher than that of difficult category materials (*M* = 0.68, *SD* = 0.23). These two main effects were further qualified by a significant interaction between the learning strategy and material difficulty, *F* (1, 58) = 6.437, *p* = .014, $\eta_{p}^{2}$ = 0.100. For the difficult category, the delayed JOL group (*M* = 0.75, *SD* = 0.21) performed significantly better than the restudy group (*M* = 0.60, *SD* = 0.24), *F* (1, 58) = 6.836, *p* = .011, $\eta_{p}^{2}$ = 0.105, while there was no significant difference between delayed JOL (*M* = 0.86, *SD* = 0.12) and restudy group (*M* = 0.82, *SD* = 0.16) when studying the simple category, *F*(1, 58) = 0.918, *p* = .342, $\eta_{p}^{2}$ = 0.016 (see Figure 6).

### 4.3. Discussion

Experiment 2 was designed to examine the forward effects of item-based delayed JOLs on category learning and the role that material difficulty played in these effects. We found that item-level delayed JOL also promoted subsequent learning of new material in category learning tasks, contrary to previous findings, suggesting that cue-only JOL may be necessary for this facilitative effect. Moreover, for the category learning tasks, there was also an interaction between the learning strategy and material difficulty, which was in line with our previous hypothesis, indicating that the promotion effect of the delayed JOL strategy on new learning was more evident for items with greater difficulties. That is, participants may have realized that some examples were easy to classify, but others were more difficult, which made them realize that their learning was not as successful as they had expected. This experience of failure with difficult examples leads participants to exert in more effort and adopt more effective retrieval and coding strategies to prepare for subsequent learning (Bransford and Schwartz 1999; Kapur 2011). Collectively, these findings provide further evidence that metacognitive judgments can improve learning performance at deeper levels of cognitive processes and is not limited to memory tasks.

## 5. General Discussion

Using three experiments, the present study tested whether delayed JOL as a learning strategy could improve performance in subsequent learning of new material, both in a word pair memory task and in category learning. This not only provided empirical support for the forward effect of delayed JOL, but also further refined the conditions under which this facilitation effect applies.

Making metacognitive judgments can positively affect future memory performance (Soderstrom and Bjork 2015; Witherby and Tauber 2017). Delayed JOL is an important form of metacognitive judgment, and to examine learners’ subjective monitoring of their own learning, research participants are often asked to assess their likelihood of success on future tests. Whether learning single words or word pairs, if the process of delayed JOL involves implicit retrieval of the target item (e.g., presentation of only the stem or only the target word), then it can lead to a forward facilitation effect. This is consistent with the results found by Kubik et al. (2022) and perhaps also helps to explain the ineffectiveness of the results found in Experiment 1 by Lee and Ha (2019). Lee and Ha presented cues (paintings) and target items (artists’ names) simultaneously, which may have caused the learners to place themselves in a state similar to restudy without sufficient metacognitive assessment. In other words, the participants were not encouraged to retrieve the target words according to the cue items when making JOLs; therefore, the retrieval practice step was missing. Moreover, the meta-analysis results of Rhodes and Tauber (2011) showed that delayed JOL improved memory when presented the cue words only instead of a word pair, which was the only condition that allowed learners to make diagnostic attempts at long-term memory retrieval before the output of JOLs (Dunlosky and Nelson 1992; Weaver and Kelemen 2003; Rhodes and Tauber 2011). There also seems to be a similar effect in terms of source memory monitoring, where participants may try to retrieve an item’s source when a Judgment of Source (JOS) is delayed and only items are available, whereas they do not engage in source retrieval when the complete source–item pair is presented (Schaper et al. 2022, 2023).

In addition, Metcalfe and Finn (2008) found that learners experience at least two stages in the process of delayed JOL: the stage of familiarity judgment and the stage of target retrieval. Only after the target retrieval stage will the memory trace be deepened, thus improving the learning performance. In our study, only cue words were presented in the delayed JOL stage so that the participants could predict and judge the possibility of correctly recalling the target word in the future test. This process enabled learners to adopt more effective retrieval or coding strategies in subsequent learning according to the assessment of their learning performance. Therefore, compared with the restudy strategy which did not involve retrieval practice, the delayed JOL of only presenting cue words could significantly promote the learners’ subsequent learning of new material.

In Experiment 1A, the forward effect of delayed JOL was found to occur in materials of medium difficulty. Experiment 1B and Experiment 2 further explored the effect of material difficulty on the forward effect of delayed JOL by manipulating word–pair relevance in the memory task and the number of dimensions in the category task, respectively. The results showed that the forward effect of delayed JOL was present only for material of medium or high difficulty, and not for very easy to learn tasks. The failure-encode-effort theory suggests that failure to retrieve in the prior interim task leads participants to realize that their memories are more deficient than they thought they would be, which motivates learners to commit more effort to encode new information in order to narrow the perceived gap (Cho et al. 2017). Previous studies have shown that retrieval failures or errors made in previous tests can encourage encoding in subsequent study phases (Potts and Shanks 2014; Soderstrom and Bjork 2014; Yang et al. 2017).

In addition to enhancing encoding effort, the retrieval–effort theory proposed by Cho et al. (2017) may also help us to understand that the forward effect found in the current study is more obvious in materials with higher levels of difficulty. This theory hypothesizes that retrieval failure in a prior interim task causes learners to be dissatisfied with their learning performance. This dissatisfaction motivates them to exert more effort in subsequent learning to retrieve new information, and that greater retrieval effort yields better recall performance (Cho et al. 2017; Yang et al. 2018). Evidence for the retrieval–effort theory comes from Yang et al.’s Experiment 3. The experiment consisted of two groups, a test group and a restudy group, using the painter’s painting style and works as the experimental material. In the interim test of the last list, Yang et al. assessed their participants’ retrieval effort by measuring how much time they spent classifying the paintings. The result showed that the interim test group spent more time classifying the paintings than the restudy group, which was consistent with their hypothesis (Yang et al. 2019). Thus, we might conclude that the learner’s effort and motivation play a moderating role in the forward effect. When presented with materials of varying difficulty in random order, the learners realized that learning the difficult material was not been as successful as they expected, and this experience of failure led them to exert more effort into encoding, using more effective retrieval strategies, or both, to prepare for subsequent learning.

For cumulative test scores, we found in Experiment 1A that the JOL group outperformed the restudy group. This result did not surprise us, as many previous studies have shown that making item-by-item JOLs can retroactively alter memory itself, a phenomenon known as the memory reactivity effect (Tauber et al. 2015; Witherby and Tauber 2017; Double et al. 2018; Janes et al. 2018; Tekin and Roediger 2021; Li et al. 2022). A positive reactivity theory proposes that making item-by-item JOLs may motivate participants to exert greater encoding effort, use more effective encoding strategies, and engage in more elaborate processing, leading to improved retention and a positive reactivity effect (Mitchum et al. 2016; Li et al. 2022). The results of Experiment 1B suggest that the advantage of delayed JOL is only demonstrated when the learning material is of some difficulty, perhaps because the easy items are not challenging for most learners and require little retrieval effort for them to learn, so retrieval practice seems to play a minimal role in the process. For the difficult items, the desirable difficulty theory suggests that activities that are more difficult and require more effort to retrieve are better for performance than those that are relatively easy to retrieve (Bjork and Bjotk 1992). Bjork R. A and Bjork E. L. proposed the concepts of retrieval strength and storage strength and believed they could be improved by increasing retrieval difficulty to promote long-term retention of memory. If the ability to retrieve and store an item is weak, it is relatively difficult to retrieve the item. In other words, the higher the retrieval ability and storage intensity of the item after successful retrieval, the better the learning performance will be. Thus, the delayed JOL strategy that involves covert retrieval is significantly more effective than the restudy strategy. However, slightly different from previous studies, it is worth noting that since List 3 was tested once in the current study, the performance of the two groups on the cumulative test may have been affected by the testing effect of List 3 and not only the effect of the delayed JOL. Nevertheless, because both groups of participants were affected by the interim test in List 3, there were still significant differences on the final cumulative test, which we believe may also reflect, to some extent, the different effects of the two learning strategies on the learned material.

For educational practice, the discovery of the forward effect of delayed JOL provides a feasible learning strategy for practical learning. In the traditional view, tests are often used to measure students’ knowledge by using a standardized measure to evaluate the students’ achievements (Roediger and Butler 2011). However, in actual educational practice, frequent testing is not generally accepted. Some educators believe that tests are time-consuming and laborious, which not only occupy teachers’ time to prepare, but also cause some students to suffer from test anxiety, depression tendencies, decreased learning motivation, and poor performance due to frequent tests (Hancock 2001). However, together with the results of previous studies, this study shows that delayed JOL has great potential to improve learning performance not only on old and new material, but also on memory and categorical tasks. Therefore, teachers can introduce metacognitive strategies to students throughout the daily teaching process so that students can use cue-only metacognitive judgment to prepare for subsequent learning, especially when the learning material is difficult. For example, a teacher may provide students with metacognitive questions about learned concepts before learning new concepts.

## 6. Limitations

The first limitation of this study is that its participants were all college students from the same school; therefore, the population structure of participants was relatively lacking in diversity, and the representation was insufficient to generalize the results. In particular, the students at this age have a good memory, have received a good education, and may use particular strategies proficiently in learning while learners at other ages may adopt different memory methods. Thus, in future research, researchers can choose primary and secondary school students as research groups to observe whether there is a difference in the results.

Second, our study used word pairs and artificial concept materials to explore the learners’ performance. Although these have enriched the experimental materials to some extent, it is not enough to prove that the forward effect of delayed JOL is a general phenomenon in learning. Future research could examine whether the forward effect of delayed JOL is robust in other learning materials or tasks.

Third, this study demonstrates that delayed JOL as a learning strategy can improve subsequent performance in new material and examines the necessary role of retrieval practice in this process. Based on the covert retrieval hypothesis, although delayed JOL involves covert retrieval and testing involves overt retrieval, the two learning strategies in paired-associate learning paradigms often produce similar benefits relative to restudy on delayed recall (Smith et al. 2013; Putnam and Roediger 2013). For example, Putnam and Roediger’s study (Experiment 3) compared restudy, covert retrieval, and overt retrieval and found that covert and overt retrieval yielded similar levels of recall, with both groups of participants recalling better than the restudy group. However, the truncated retrieval hypothesis challenges this view by suggesting that, relative to overt retrieval practice, people exert less effort into retrieval during delayed JOLs and thus truncate their retrieval attempts prematurely (Son and Metcalfe 2005; Tauber et al. 2015; Tekin and Roediger 2021). Tekin and Roediger (2021) examined the effect of delayed JOLs on subsequent recall by comparing them to restudy, overt retrieval, and several other delayed JOL conditions. Their results showed that providing delayed JOLs reliably improved recall relative to restudy, but providing only delayed JOLs did not produce similar benefits relative to overt retrieval. Therefore, a strict covert retrieval hypothesis may not explain the promotion effects of delayed JOLs. Although this study provides some support for the covert retrieval hypothesis, it does not show that the effects of covert and overt retrieval are identical because they were not directly compared to the test group. Future studies could test the truncated retrieval hypothesis by directly comparing the delayed JOL with the test. In previous studies of the forward effects of tests, researchers have proposed the context-change theory, strategy-change theory, activation facilitation theory, test-expectancy theory, and others. Considering that the delayed JOL and test have a certain common basis, whether these theories are also applicable to the delayed JOL needs to be further tested (Sundqvist et al. 2012; Akdoğan et al. 2016).

Fourth, the results of previous related studies support the conclusion that cue-only JOL encourages learners to make retrieval attempts, both in terms of making delayed JOLs for the studied material (e.g., Rhodes and Tauber 2011) and in terms of the forward effect of delayed JOLs (e.g., Kubik et al. 2022). While the present study supports this conclusion by showing that delayed cue-only JOLs had a positive effect on word-pair and category learning, it is important to note that the effect of delayed JOLs with full information cannot be fully ruled out since they were not directly compared in this experiment.

Finally, this study found that material difficulty was one of the factors affecting the forward effect of delayed JOLs, but we did not directly measure and collect data about the students’ motivation. Although previous hypotheses, such as the failure-encoding-effort theory and the retrieval-effort theory, have received empirical support, providing data such as response times may explain our results more intuitively. Future research could investigate the influence of motivational factors on the forward effect by measuring the self-paced learning time of participants on new material.

## 7. Conclusions

Through three experiments, this study concluded that the forward effect of delayed JOL not only exists in word pair learning, but it can also promote subsequent performance in new learning categories. The promoting effect was observed only for materials of medium or high difficulty.

## Data Availability

All data analyzed in the current study are available from the corresponding author upon reasonable request.

## Supplementary Materials

The following supporting information can be downloaded at: https://www.mdpi.com/article/10.3390/jintelligence11060101/s1, Figure S1: The category learning materials used in Experiment 2; Table S1: Word pairs used in the experiment 1A; Table S2: Word pairs used in the experiment 1B.
