# Supplementary material for: The Forward Effect of Delayed Judgments of Learning Is Influenced by Difficulty in Memory and Category Learning
Source: J Intell. 2023 May 25;11(6):101. doi: 10.3390/jintelligence11060101 (PMC10299460; doi:10.3390/jintelligence11060101)
Supplement: Supplementary file 1 [file jintelligence-11-00101-s001.zip › jintelligence-2298673-supplementary.pdf]

Supplemental Information

Table S1

Word pairs used in the experiment1A

list1	list2	list3
**Formal experiment**		
电影——公斤	钥匙——瀑布	泥浆——编辑
生日——篱笆	孔雀——档案	态度——石油
自然——公安	协会——速度	蜻蜓——皮球
显示——战争	海峡——扇子	机会——卫生
轨道——透镜	字典——日光	电灯——手绢
杯垫——火车	说明——小时	整体——学者
论文——季风	礼物——高楼	金子——树林
瓶口——蝴蝶	城镇——立场	运动——主管
飞鸟——篮子	风险——形象	马桶——窗户
外衣——画家	武器——消息	萝卜一一肚皮
货币——文章	集中——散步	水池一一大米
玉米一一高山	权力一一效果	水壶一一饭盒
**Practice block**		
人物一一方法	工人一一现实	积雪一一农历
烟囱——篱笆	区域——规律	路线——医院

Table S2

Word pairs used in the experiment1B

list1	list2	list3
**Formal experiment**		
Easy		
保健——卫生	公里——长度	案件——犯罪
珍珠——贝壳	机械——零件	交易——股票
司机——汽车	信件——邮票	意志——品质
店员——服务	报刊——杂志	衬衣——领带
人生——命运	大意——疏忽	公式——方程
挖掘——潜力	旅客——列车	火海——燃烧
Difficult		
树干——手表	轮船——大肠	翅膀——苹果
石板——眼皮	客车——榕树	松鼠——钉子
鼠标——火柴	儿童——化石	灯笼——水库
礼堂——山峰	台阶——蜻蜓	肩膀一一茶馆
孔雀——宝剑	河流——牙齿	报社一一板凳

电视一一葫芦	木棍一一烟斗	刀片一一窗户
**Practice block**		
Easy		
彩虹一一祥云	眼泪一一情绪	羽毛一一企鹅
Difficult		
银行——轮船	气球——桥梁	岩石——器官

Figure S1

The category learning materials used in Experiment 2

List1

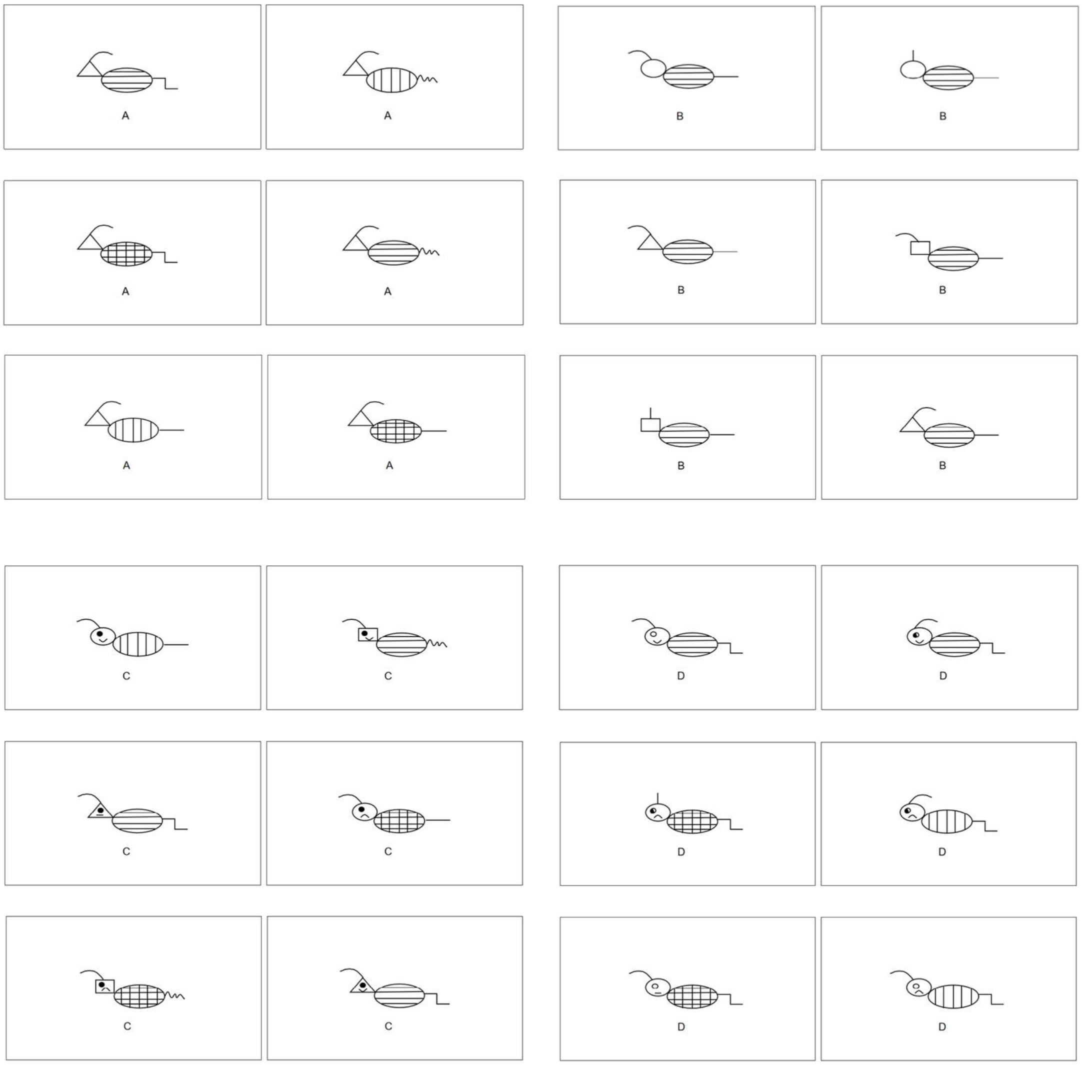

List2

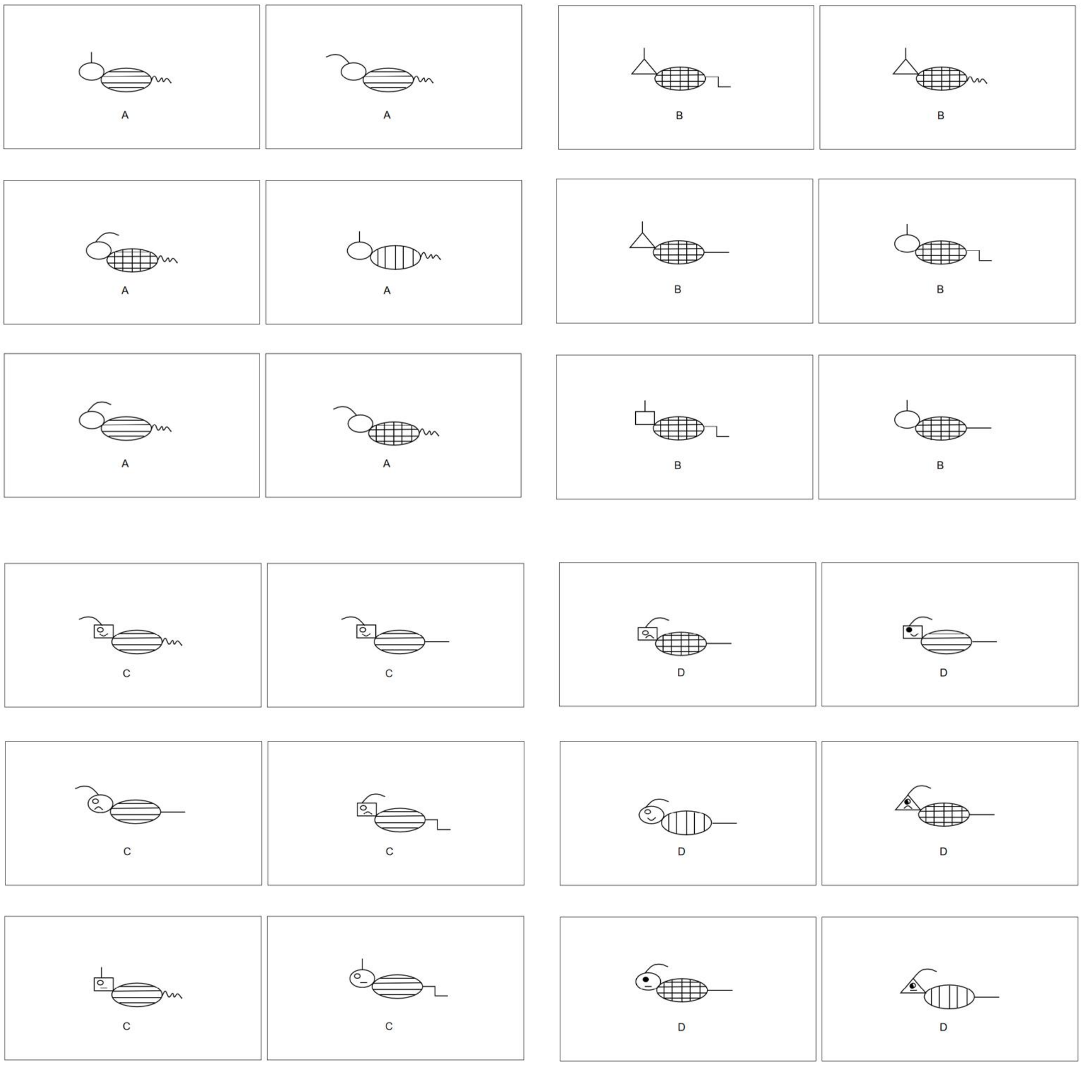

List3

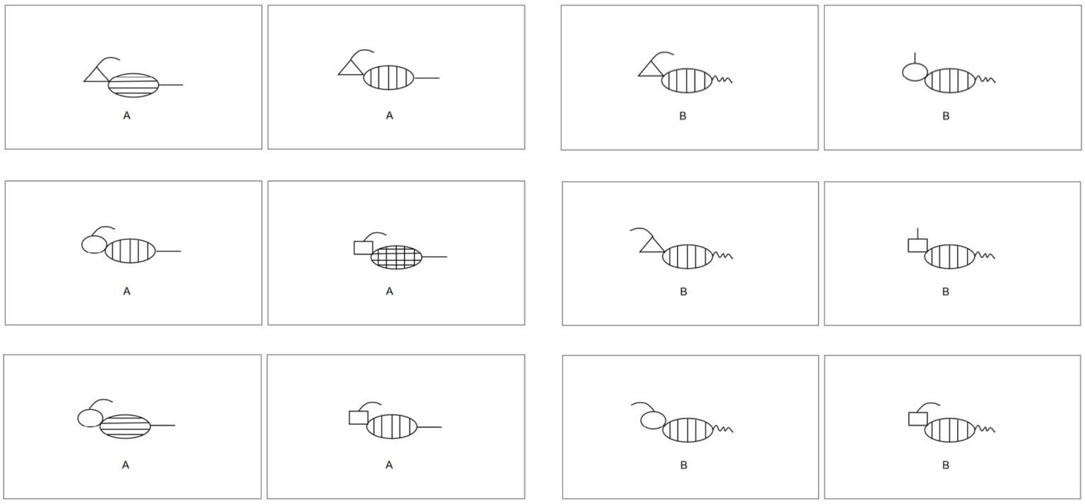

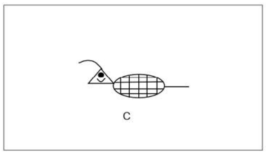
C
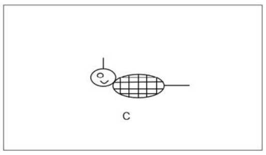
C
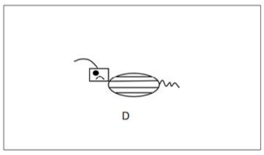
D
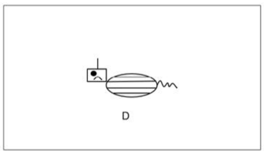
D
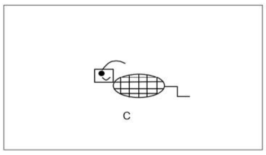
C
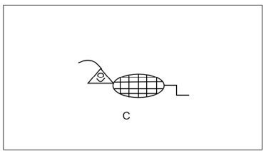
C
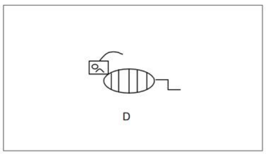
D
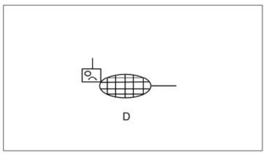
D
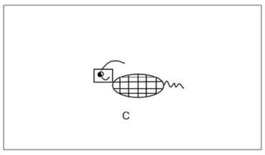
C
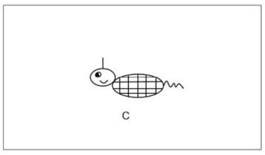
C
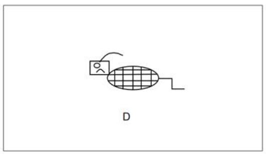
D
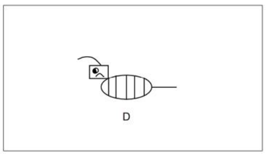
D
